# Sensory Neuronopathy and Autoimmune Diseases

**DOI:** 10.1155/2012/873587

**Published:** 2012-01-19

**Authors:** Alberto R. M. Martinez, Marcelo B. Nunes, Anamarli Nucci, Marcondes C. França

**Affiliations:** Department of Neurology, Faculty of Medicine, University of Campinas (UNICAMP), 13083-887 Campinas, SP, Brazil

## Abstract

Sensory neuronopathies (SNs) are a specific subgroup of peripheral nervous system diseases characterized by primary degeneration of dorsal root ganglia and their projections. Multifocal sensory symptoms often associated to ataxia are the classical features of SN. Several different etiologies have been described for SNs, but immune-mediated damage plays a key role in most cases. SN may herald the onset of some systemic autoimmune diseases, which further emphasizes how important the recognition of SN is in clinical practice. We have thus reviewed available clinical, neurophysiological, and therapeutic data on autoimmune disease-related SN, namely, in patients with Sjögren's syndrome, autoimmune hepatitis, and celiac disease.

## 1. Overview of Sensory Neuronopathies

### 1.1. Definition and Historical Aspects

Sensory neuronopathies or ganglionopathies (SNs) constitute a specific subgroup of peripheral neuropathies characterized by primary and selective dorsal root ganglia (DRG) neuronal destruction [1–3]. Degeneration of DRG “T-shaped” neurons and their projections, both central and peripheral, often results in a multifocal pattern of sensory deficits [4, 5]. This is in contrast to the usual length-dependent pattern found in most polyneuropathies. Although relatively rare, SN should be included in the differential diagnosis of predominantly sensory or ataxic neuropathies. On clinical grounds, recognition of SN is important because it reduces the number of etiologies to be investigated and also because some of these SN-etiologies are amenable to effective treatment [6].

SNs were first described in 1948 by Denny-Brown in two patients with bronchial carcinoma that developed acute-onset predominantly sensory peripheral neuropathy involving arms, legs, face, and tongue [7]. Postmortem analysis showed a massive and selective destruction of DRG neurons. This description also pointed for the first time to the possible association between SN and neoplasia, which later proved to be true. In the next years, Dyck et al. described DRG histological damage in patients followed up at the Mayo Clinic due to peripheral neuropathy of unknown etiology [8]. Since then, several mechanisms have been proposed to explain DRG destruction in patients with SN, including genetic predisposition, drug-related toxicity, infections, and immune-mediated damage [2, 9]. The latter mechanism probably takes part in most patients with SN, and several autoimmune systemic diseases have been associated with SN. In this setting, our scope is to review clinical, pathophysiological, and therapeutic aspects of SN related to Sjögren's syndrome (SS), celiac disease (CD), and autoimmune hepatitis. These are the most frequent autoimmune diseases associated with SN.

### 1.2. Epidemiology

SNs are traditionally considered rare disorders, but underdiagnosis is certainly a problem [1]. Most available epidemiological data refer to paraneoplastic and SS-related SN [2, 9, 10]. Overall, paraneoplastic neurological syndromes are uncommon and affect nearly 0.01% of all oncologic patients [10]. SN is the most frequent paraneoplastic syndrome and represents around 20% of all paraneoplasia in a recent European report [11]. SS is the most frequent immune-mediated disease related to SN [12]. Some authors estimate that 10% of all patients with SS will ultimately develop a SN. Unfortunately, 50% of the cases with SN are still labeled as idiopathic [1, 13, 14].

### 1.3. Pathophysiology

Capillaries that supply DRG neurons have a leaky basement membrane, which enable the passage of inflammatory cells, toxins, and proteins. This explains why DRG neurons are vulnerable to such distinct mechanisms of damage [9]. In immune-mediated SN, most available data support the concept of direct inflammatory damage to DRG neurons mediated by CD8 T lymphocytes [3, 5, 9, 14–16]. Humoral dysfunction seems to play a minor role in most forms of SN, but anti-GD1b antibodies were associated to SN in cell and animal-based models [17, 18]. In addition, rare patients with SN present high serum titers of anti-GD1b [19].

 Interestingly, immune mechanisms have been lately described in patients with idiopathic SN as well. We have recently found high IL-17 expression combined with reduced IL-27 expression in CSF lymphocytes. There was also an increase in CD8 lymphocyte proportion, but not CD4, in the blood and CSF of those patients with disease duration smaller than 5 years when compared to those with duration longer than 5 years [20].

### 1.4. Clinical Aspects

SN manifestations are often disabling, but the specific symptoms depend on the type of involved fibers [9]. Deficits are often multifocal and extend to both proximal and distal regions of the limbs; all sensory modalities—pain, temperature, sense position, and vibration—may be compromised during disease course [2]. Large myelinated fibers that convey sense position and vibration are predominantly damaged in SN. This leads to gait ataxia and widespread arreflexia [1, 2]. Some patients present pseudoathetotic hand movements. Whenever small-and medium-sized neurons degenerate, pain and burning allodynia also appear [1, 2]. Motor system examination is usually unremarkable. Nystagmus is not frequent, but autonomic dysfunction may be found. There are reports of tonic pupils, orthostatic hypotension, gastrointestinal symptoms, and erectile dysfunction [4].

 There are also some etiology-specific findings such as limbic encephalitis that is characterized by recent memory deficits, behavioral changes, and seizures and are found in 20–30% of patients with anti-Hu paraneoplastic syndrome [10]. Friedreich's ataxia shows typical feet deformities, severe kyphoscoliosis and, square-wave jerks [21].

 Clinical course may also be useful to differentiate autoimmune/idiopathic causes from paraneoplastic ones. Chronic course is more common in idiopathic disease whereas an abrupt onset is typically seen in paraneoplastic or autoimmune SN [2, 3]. In contrast to other immune-mediated neuropathies, SN hardly presents a remitting-recurrent course.

### 1.5. Diagnostic Tests

SN has a distinctive clinical picture, but diagnosis often relies on complementary workup. This includes nerve conduction studies, neuroimaging, and pathological analyses.

#### 1.5.1. Nerve Conduction Studies (NCSs)

NCS are the most useful tests in the evaluation of suspected SN [6]. NCSs classically show a sensory neuropathy without a distal worsening gradient towards the legs. Sensory NCSs reveal widespread reduction of sensory action potential amplitudes combined with normal conduction velocity. Asymmetric responses are typical of SN. Motor NCSs are often normal, but at least 18% of patients show reduced amplitudes of compound muscle action potentials, especially at peroneal and tibial nerves [6]. Electromyography is usually normal as well. However, some patients present an abnormal recruitment pattern of motor units that is especially evident during maximal activation. Blink reflex study is another useful tool because it may help to differentiate paraneoplastic versus non-paraneoplastic SN [22].

#### 1.5.2. Neuroimaging

Magnetic resonance imaging (MRI) is a sensitive technique to diagnose patients with SN, especially those with long disease duration. This is because DRG damage leads to degeneration of their central projections—gracile and cuneate fasciculi—which results inspinal cord atrophy and gliosis. Cervical spinal cord MRI scans therefore show hyperintense T2-weighted lesions at posterior columns and volumetric reduction in chronic SN [23]. The combination of such MRI findings and the typical NCS abnormalities is virtually diagnostic of SN.

#### 1.5.3. Pathology

Excisional biopsy with histological analysis of DRG is the gold standard diagnostic method for SN [5]. Despite this, it is seldom performed because it is invasive and requires trained neurosurgeons. Histological findings are neuronal loss, the Nageotte nodules, and mononuclear infiltrates. In paraneoplastic SN, immunohistochemical analysis shows intraneural IgG deposits without complement deposits [5].

Sural nerve biopsy reveals loss of large and small fibers in SN, but the pattern is similar to that found in the length-dependent neuropathies. Skin biopsy with quantification of intraepidermal nerve fiber density has been recently suggested as a useful tool. This technique shows a reduced fiber density without a distal gradient in SN [24].

### 1.6. Diagnostic Criteria

Asbury and Brown were the first to propose clinical and electrophysiological criteria for SN in the early 90s. Asbury's criteria relied upon the disproportionate sensory involvement and the non-length-dependent distribution of deficits [3, 25, 26]. Although clinically useful, these were never validated so that alternative criteria were recently published and validated by Camdessanché et al. This new proposal is a score-based table that includes not only clinical and neurophysiological data, but also cervical MRI and pathological findings [3].

## 2. Specific Autoimmune-Disease-Related SN

### 2.1. Sjögren's Syndrome

Primary Sjögren's syndrome (SS) is a systemic autoimmune disease that affects 1-2% of the population [27]. The core clinical findings are xerophthalmia and xerostomia (sicca syndrome), but visceral involvement such as pneumonitis, renal tubular acidosis, and pancreatitis also takes place [28]. Several neurological manifestations are associated with SS, including acute myelitis, neuromyelitis optica [29], and brainstem disease [1]. Peripheral nervous system is damaged in about 50% of those patients with SS-related neurological disease [2]. SS-related peripheral nerve damage may present as cranial neuropathy (trigeminal), *mononeuritis multiplex*, radiculoneuropathy, painful small fiber neuropathy, autonomic neuropathy (with anhidrosis), and SN [30].

Recent data indicate that 15–39% of all patients with SS-related neuropathies actually have SN [2]. SN usually antedates the diagnosis of SS. Most affected patients are in their 60s or 70s (mean age of 64.9 years) and present subacute disease over weeks or few months [31]. In SS-related SN, sensory disturbances are often unilateral or strikingly asymmetric. Upper limbs are predominantly affected, but the trunk, face, or lower limbs are often involved as well [2]. Sensory ataxia and widespread arreflexia are conspicuous findings, but pain or painful dysaesthesias are only found in 50% of these patients. Trigeminal involvement has been reported in 30% of the subjects and pseudoathetoid hand movements in a smaller proportion of cases. Dysautonomic symptoms are frequent, contribute to overall disability and may present as hypo/anhidrosis, tonic pupils, and gastrointestinal and cardiovascular dysfunction [2, 30].

Nerve conduction studies typically show widespread reduction of sensory nerve action potential amplitudes, but no significant reduction of conduction velocity. In some patients, abnormalities are asymmetrical and median nerves may be more severely compromised than sural nerves. Motor conduction studies and needle EMG are often normal. Somatosensory-evoked responses reveal abnormal central conduction times which are probably due to the degeneration of dorsal columns in the spinal cord [3]. This central damage is also revealed by spinal cord MRI, which presents T2 hyperintense lesions affecting both gracilis and cuneatus fasciculi. Mori et al. have shown that such MRI abnormalities correlate with clinical dysfunction in SS-related SN [3, 30, 32]. There is no serum marker for SS-related SN, but anti-Hu antibodies are sometimes useful to distinguish it from the closely related paraneoplastic form of SN. In addition, anti-Hu seropositivity suggests a paraneoplastic etiology even in those subjects with an autoimmune disease diagnosed [33].

 The pathological substrate of SS-related SN is a ganglionitis mediated by T CD8 lymphocytes. Recent evidence indicates that humoral dysfunction plays a minor role. Although the precise pathogenic cascade and the target antigen are still unknown, upregulation of proinflammatory cytokines—particularly tumor necrosis factor alpha (TNF*α*)—is a key event [34].

 There are no controlled trials devoted to the treatment of SS-related SN. Most available data about therapy rely upon small series or retrospective analyses [35]. Chen et al. reported dramatic and sustained improvement in 2 out of 4 patients after five to nine sessions of plasma exchange [36]. In another study, 4 out of 5 patients with chronic disease showed a remarkable improvement after three cycles of IVIG (0.4 g/kg for 5 consecutive days) given at 2-week intervals [37]. In contrast, Rist et al. reviewed the use of IVIg in peripheral neuropathies related to SS and found that IVIG was not as effective in the treatment of SS-related SN as it was for sensory-motor neuropathies [38]. Rituximab was lately shown to be effective as an IVIG-sparing agent in a patient with SS-related SN that responded to IVIg [39]. The TNF*α* antagonist infliximab (3 mg/kg) was reported as beneficial in a single patient with refractory SN [40]. In our own experience, azathioprine (2-3 mg/kg a day) also proved effective in occasional patients.

### 2.2. Autoimmune Hepatitis

Autoimmune hepatitis (AIH) is a chronic inflammatory liver disease of unknown etiology. Environmental triggers, failure of immune tolerance mechanisms, and genetic predisposition probably collaborate to induce T-cell-mediated attack upon liver antigens, leading to a progressive necroinflammatory and fibrotic process. Women are affected more frequently than men (3.6 : 1), but the disease is seen in all ethnic groups and ages [41]. The diagnosis of AIH relies upon specific clinical and laboratory criteria and the exclusion of other viral, genetic, and toxic conditions [41, 42].

 The association of AIH with SN was first reported in 1993 by Merchut et al. that described a woman with AIH that developed progressive non-length-dependent ataxic neuropathy, predominantly affecting the arms. Paresthesias and ataxia failed to improve with immunosuppression [43]. Liedholm et al. in 1994 reported a woman with chronic persistent hepatitis that developed sense position impairment in the arms after the acute phase of hepatic illness. She presented mild improvement with the therapy for hepatic disease [44]. Magy and colleagues then reported a 40-year-old woman with AIH and paresthesias that rapidly evolved into severe gait ataxia and global arreflexia. She was treated with IVIg (0.4 g/Kg/d—5 sessions), but symptoms did not improve. Prednisolone was then started, but resulted in only partial benefit [45]. At least two additional patients were reported since then, but detailed clinical data are not available [1, 13].

 If we consider that both SN and AIH are unusual conditions, these previous reports probably indicate that there is a real association between them. However, with the available data, one might speculate only whether there is a cause-and-effect relationship or that both diseases are organ-specific expressions of an underlying widespread immunological disturbance. Further studies with larger series are certainly needed to clarify this issue and to delineate the clinical profile of AIH-related SN.

### 2.3. Celiac Disease

Celiac disease (CD) is an autoimmune disorder related to the ingestion of wheat gliadins or other cereal prolamins by susceptible individuals. This susceptibility is due to predisposing hereditary factors that include both HLA and non-HLA genes [46]. More than 90% of patients with CD carry the high-risk alleles HLA-DQ2 and HLA-DQ8 [47]. Prevalence of CD depends on the population studied and varies from 1 : 70 to 1 : 500 [46–49]. The lifelong incidence is 1 : 100, and any age group can be affected [46]. Population-based studies in Finland also suggest that the prevalence increases with age from 1.5% in children to 2.7% in the elderly [50–53]. Women are preferentially affected by the disease with a 2 : 1 ratio [54].

The classical symptoms of CD are chronic malabsorptive diarrhea, flatulence, iron deficiency anemia, and weight loss, but extraintestinal manifestations are also possible, such as osteopenia, aphthous stomatitis, arthritis, liver failure, and psychiatric and neurological manifestations [47–50]. In fact, small bowel involvement is not a *sine qua non *condition to establish the diagnosis of CD. Extraintestinal manifestations may precede or even occur without overt intestinal involvement.

 Neurological manifestations of CD involve both central and peripheral nervous system. They are found in 10–28% of patients with an established diagnosis of CD [55]. Central manifestations include ataxia (gluten ataxia [55]), headache, epilepsy with or without parietooccipital calcifications, encephalopathy, myelopathy, intellectual degeneration with attention/memory impairment, and stiff-man syndrome [48, 55–58]. Peripheral involvement is characterized by symmetric sensory-motor axonal neuropathy, *mononeuritis multiplex*, autonomic neuropathy, pure motor neuropathy, small-fiber neuropathy, and SN [49, 57].

 CD-related SN was recently reported by Hadjivassiliou et al. in a large series of British patients that were regularly followed by chronic neuropathies [57]. Out of 409 patients, 13% (53/409) had clinical and neurophysiological signs of SN and 17 of those (12 women : 5 men, 17/53 = 32%) had serological evidence of gluten sensitivity. Biopsy-proven enteropathy was found in 7 patients out of the 17. In this survey, CD-related SN thus accounted for 8% of all CD-related neuropathies [55, 57]. Mean age of these 17 patients was 67 years (range 47–85), and mean age at onset of sensory symptoms was 58 years. In this study, mild/moderate sensory ataxia was the usual chief manifestation of CD-related SN. In another study, Brannagan III et al. reported 8 patients with CD that developed SN but with predominant small fiber involvement [49]. These patients had non-length-dependent reduction of intraepidermal nerve fiber density, and their symptoms included asymmetrical numbness or paresthesias involving limbs, hands, feet, and face as well as mild to moderate sensory ataxia [49].

 CD is a peculiar autoimmune disease because the triggering antigen, gluten, is already known [57]. The neurological manifestations are also immune mediated, and both cellular and humoral responses take place [55, 58]. Several antibodies have been associated to neurological damage such as IgG antibodies against gliadin, IgG-deamidated gliadin peptide antibodies, IgA antibodies against endomysium, and IgA antibodies against different transglutaminases, specially the anti-transglutaminase 6 antibodies [50, 55, 56, 58]. Postmortem analyses and peripheral nerve biopsies showed lymphocytic infiltrates with perivascular cuffing as the pathologic findings [57, 58].

 Some patients had a slowly progressive course that was not modified by the classical gluten-free diet (GFD). There were occasional patients that presented disease remission or stabilization while adherent to GFD. Immunosuppressive therapy needed to be combined with GFD in order to induce remission for some refractory patients [55, 57].

## 3. Conclusion

SN has a rather typical clinical presentation characterized by non-length-dependent and exclusively sensory deficits. It may be associated to several autoimmune diseases, and sometimes SN is the first manifestation of the underlying systemic condition. SS, AIH, and CD are the autoimmune diseases most frequently associated to SN. Despite this, many issues regarding the mechanisms of dorsal root ganglia damage in the setting of systemic autoimmunity remain unanswered. Further longitudinal studies with large samples of patients are needed to delineate the pathophysiology and the better treatment options for autoimmune disease-related SN, especially in association with AIH and CD.
